# Ruggero Ceppellini: A Perspective on His Contributions to Genetics and Immunology

**DOI:** 10.3389/fimmu.2019.01280

**Published:** 2019-06-05

**Authors:** Walter Bodmer

**Affiliations:** Department of Oncology, Weatherall Institute of Molecular Medicine, University of Oxford, Oxford, United Kingdom

**Keywords:** Ceppellini, HLA, MLC, haplotype, transplantation

## Abstract

Ruggero Ceppellini, who died at the age of 71 in 1988, was one of the most stimulating and original human geneticists of his generation (1). Ceppellini's outstanding contributions to the genetics of the human blood groups, immunoglobulin allotypes and the HLA system epitomize the study of immunogenetics. By using his considerable skills and insights to unravel the interpretation of the serological data, he made significant contributions to immunology. He is remembered especially for his incisive contributions to the development of the genetics of the HLA system and its nomenclature, including, in particular, his introduction of the term “haplotype,” now widely used by geneticists throughout the world, most of whom are unlikely to be aware of its origins.

## Early Blood Group Discoveries

Born during the first world war, Ceppellini was caught up by military service in the second world war and so could not finish his medical studies until after the war had ended. During his service as a sergeant in World War II, Ceppellini was captured by the British and taken as a prisoner of war in Palestine, where the charismatic physician and human geneticist, Chaim Sheba, took him on as a medical orderly because of his medical background. Many years later, when Ceppellini was attending a human genetics meeting in Israel, Chaim Sheba greeted him as “Sergeant Ceppellini.” Perhaps it was that brush with genetics that stimulated his interest in the field and led to his appointment, through the influence of Luca Cavalli-Sforza, to a position in the Istituto Sieroterapico Milanese, a blood bank associated with the University of Milan. Cavalli–Sforza, although Ceppellini's junior by 5 years, was already becoming established as a geneticist and was a major influence on Ceppellini's future career.

In 1954, Ceppellini was invited to work in the Institute for the Study of Human Variation at Columbia University in New York, where he came under the influence of L. C. Dunn and made his first significant contribution to immunogenetics. He showed, through a careful family and population based study, that the Rh variant D^u^ was actually due to a reduced expression of D when associated in heterozygotes with the combination Cde (2).

Shortly after his return to Italy in 1959, Ceppellini made his second outstanding contribution to the blood grouping field. This was his interpretation of the Lewis b phenotype as an interaction between the secretor and Lewis genes, and his interpretation of the ABO and Lewis blood groups in terms of a form of metabolic sequence involving successive additions of sugars. His model, based entirely on a genetic interpretation of the data, showed remarkable insight and was abundantly confirmed by the studies by Morgan, Watkins, Kabat, and others of the oligosaccharide structures of these blood group determinants and the eventual identification of the two fucosyltransferase genes (3, 4).

## Malaria, Thalassaemia and the Immunoglobulin (Gm) Allotypes

In the early 1950s, Ceppellini had started a systematic study in Sardinia to correlate the distribution of thalassaemia, which was common there in the low lying villages, with the distribution of malaria that had been established in Sardinia by an extensive study of school children. Through this, he provided some of the first clear evidence of the correlation between thalassaemia and resistance to malaria, showing that its frequency was highest in those areas where the incidence of malaria had been greatest (5). His work there stimulated a long and continuing tradition of studies in human genetics in Sardinia, carried forward especially by his close friend and colleague Marcello Siniscalco.

My initial contact with Ruggero Ceppellini was established through Luca Cavalli-Sforza, in the early 1960s, because of my interest in the studies of thalassaemia and malaria as a model of natural selection in human populations. I, and my population genetics colleagues at Stanford University, invited him as a population geneticist to discuss this work. In characteristic manner, just before he was due to arrive, we received a telegram saying that unfortunately, after all, he was not able to come.

Stimulated by his contact with Henry Kunkel during his time in New York, Ceppellini took up the study of the Gm types. Kunkel was a pioneer of the study of the single immunoglobulins produced by myelomas, while Grubb had shown in 1956 (6) that there were inherited serologically detectable differences in the immunoglobulins, which were called Gm allotypes, G for immunoglobulin G and m for marker. By the early 1960s, the basic two chain structure of the immunoglobulins and the distinction between constant and variable regions had been elucidated, so that it became clear that these allotypes were inherited variations in the IgG heavy chain constant regions.

In a major comprehensive and inciteful review of the Gm allotypes, published in Italian in the proceedings of the 1966 meeting of the Italian Genetics Association (7), Ceppellini provided what was at that time the clearest interpretation of the Gm types as a complex genetic system. This was in some ways analogous to the Rhesus blood group system as interpreted by R. A. Fisher, on which he had published earlier with L. C. Dunn. His interpretation was in terms of “haplotypes” (an expression first used in this paper), which determined different combinations of Gm types, and their varying frequencies in different populations. He appreciated the possibility of the creation of new haplotypes by recombination between existing haplotypes and extended some of the formulae, which I had developed for the analysis of two locus-linked systems, to the estimation of haplotype frequencies. This notable paper is hardly ever quoted because of its publication in Italian in a more or less inaccessible journal. It is also, to my mind, odd that, as far as I am aware, this is his only publication on the Gm types. Perhaps that is because it was at this time that he started on his major interest in what became the HLA system.

## HLA, Haplotypes, Cellular Assays, and Monoclonal Antibodies

### Early White Cell Agglutination Serology

Jean Dausset pioneered the testing of sera from multiply transfused patients against white blood cells from arbitrary volunteer donors using an agglutination reaction. His aim was to establish whether these reactions could be interpreted to define inherited blood group like determinants on white rather than red blood cells. The initial results, not surprisingly in retrospect given the now known complexity of the HLA system, were very confusing.

Ceppellini suggested to Dausset in 1956 that he should compare the reactions on white cells from pairs of identical (monozygous or MZ) twins with those on pairs of non-identical (dizygous or DZ) twins. Then, if the agglutination reactions reflected inherited determinants, all the reactions should, subject to experimental error, be the same on each of the MZ twin pairs, while this would not necessarily be the case for the DZ twins. Dausset and Brecy published a short note in Nature in 1957 (8) confirming this prediction, following which, Dausset described in 1958 a putative first antigen, which he called MAC (9), and for which he shared the Nobel Prize in 1980.

The serology of the white blood cell antigens did not, however, progress any further until Jon van Rood and Rose Payne, independently in 1958, showed that sera from multiparous women contained antibodies against the white cells of their offspring that could be detected by agglutination assays. These were produced by fetal-maternal stimulation, just as in the case of the Rh blood groups and, being limited to the difference between mother and father, were much less complex than sera obtained from multiply transfused individuals. By the early to mid 1960s, Ceppellini had joined the initial group of workers in this field. In the first of the International Histocompatibility Testing workshops, organized by Bernard Amos in 1964, Ceppellini played a major role in analyzing the data and promoting the need for improved reproducibility of the testing techniques as well as for inter-laboratory comparisons of results. [For details of the early history of the HLA field, as described by its pioneers, see (10)].

As a geneticist, Ceppellini appreciated the importance of family studies, and so organized, with his colleagues, the third International Histocompatibility Testing Workshop in Turin in 1967 around the theme of a family study. While by that time it was clear that there were at least two, probably closely linked, loci for the white blood cell determinants being described, this had not been definitively established by family studies. He provided the families and we came with our sera and different technologies to test white blood cells from his family members. Ceppellini expressed to the press his amusement at seeing an erstwhile mathematician sitting at the bench looking down a microscope. The aim of the collaborative on site study was to see whether all the types being defined by the different participating laboratories were inherited together—and they were!

This, then, was the first clear-cut establishment of the HLA system as a set of closely linked genes inherited together in a way that was analogous to the Rhesus blood groups and immunoglobulin Gm allotypes.

### Haplotype

It was notably at this workshop in 1967 that Ceppellini first really introduced the term “haplotype,” though he had used it the previous year in the Italian Gm allotype review, as already mentioned. His description was as follows:

“If a new term can be introduced without increasing confusion, it is suggested to substitute phenogroup with haplotype (haploid, from α*πλ*óoς, single); in fact, the name should convey the concept that the haplotype is not an observed phene and corresponds to the product of a single gene dose.” My interpretation of this, as given in Cavalli-Sforza and Bodmer [1971, though written in 1969, (11)] was: “**haplotype** (from haploid genotype) for the combination of genetic determinants that leads to a set of antigenic specificities which is controlled by one chromosome and so inherited in coupling.”

The term was originally conceived in the context of a tightly linked cluster of alleles in strong linkage disequilibrium and before the advent of DNA based technology with its almost unlimited number of polymorphisms. However, it can clearly be generalized to refer to the set of variants to be found on any given stretch of DNA on one of the two homologous chromosomes in an individual. That DNA could extend from as little as a single exon, within which there is more than one variable position, to a whole chromosome. The concept therefore becomes vague unless it is related to a defined stretch of DNA.

### Skin Graft Survival and Blood Group Incompatibility

There was an obvious interest in establishing whether the newly identified white blood cell determinants were histocompatibility antigens in the sense of being responsible for graft rejection when not matched. Early data had suggested this was the case. Ceppellini and colleagues exchanged grafts between sibs, parents and offspring, and unrelated individuals in a systematic design. Through a collaboration with van Rood, they showed that skin graft survival times were longer when individuals were matched for the groups defined by van Rood's leukocyte agglutination assay than when they were not (12, 13).

### Mixed Lymphocyte Culture (MLC) Reaction

The Mixed Lymphocyte Culture (MLC) reaction, in which lymphocytes from different individuals when cultured together stimulate a mutual mitotic blast response while lymphocytes from the same individual do not, was discovered by Bach, and independently by Bain, in 1964. This creation of a sort of *in vitro* model for homotransplantation at the cellular level intrigued Ceppellini sufficiently to lead him to invite Fritz Bach to his laboratory to demonstrate his test. Following this, Ceppellini's group developed a “one way” MLC, in which only one of the pair of lymphocytes in a co-culture was able to respond. They then showed that some sera containing anti HLA antibodies were able to block the MLC reaction (14, 15). Ceppellini's group just missed making the important observation made by Bach and Amos (16) that MLC reactions associated precisely in families with the then serologically defined leukocyte antigens.

This discovery, however, was surely stimulated by Ceppellini's discussion of genetics with Fritz Bach. Van Rood's group was the first to define serological reactions correlating with MLC reactivities, using a rather cumbersome technique involving inhibition of MLC based on Ceppellini's discovery. This laid the foundation for the discovery of the HLA – DR and other Class II determinants using simpler B cell specific serological techniques with the same sources of sera that were used to define the HLA -A, B and C antigens. It is these anti-HLA-DR and related antibodies that explain the MLC inhibition that Ceppellini's group had first observed (see 10 for further details).

### Monoclonal Antibodies, Disease Association, and Nomenclature

Ceppellini became one of the first members of the Basel Institute of Immunology in 1970 and so was amongst the first to realize the importance of monoclonal antibodies, in particular in their application to the HLA system. It was one of his antibodies, produced with Massimo Trucco, that was first used in an International HLA Workshop in 1980. Ceppellini embraced the concept that monoclonal antibodies were the analytic tools of the future and moved from the Basel Institute to Roche as a scientist with the specific objective of producing human monoclonal antibodies. He was, as I recall, quite disappointed in the apparent lack of interest of the Roche pharmaceutical company in the work produced by the outstanding institute that they had created and supported.

In the late 1960s, Ceppellini was, not surprisingly given his background of work on thalassaemia and malaria in Sardinia, one of the first to promote the idea of looking for associations between HLA variants and diseases. It was a follow up of his work in Sardinia that first provided substantial evidence for a role for HLA in malaria resistance (17).

Ceppellini had remarkable insight not only into the genetics and biology of the HLA system but also into the quantitative aspects of its interpretation, as evidenced, for example, by his analysis of the Gm haplotypes. This brought us together in a joint publication from the 1970 International Histocompatibility Testing Workshop on the formal theory of testing the fit of two- and three-locus models of the serological data on HLA at that time and on the analysis of segregation patterns in families (18). My wife, Julia, and I remembered vividly his stay with us in California in 1970 where we finished writing the paper. He had broken his arm skiing, his favorite sport, and he was as lively as ever, but nevertheless, a broken arm did to some extent inhibit his speech! Ceppellini had, much earlier, made a significant contribution to what became a classical method for estimating gene frequencies in a random mating population using iterative gene counting (19).

Ruggero Ceppellini was one of the only professionally trained geneticists among the early workers in the HLA field, apart from myself. Through this we developed a rapport and friendship over a period of more than 20 years.

His clear thinking and forceful contributions to discussions of the WHO International Nomenclature Committee meetings helped enormously in the development of a rational HLA nomenclature based on a proper understanding of the genetics. Ceppellini shares with me and Jon Van Rood the responsibility for the HLA-DR nomenclature and so, eventually, DP and DQ.

### Conclusion

Ruggero Ceppellini unfortunately suffered from periods of depression during the 1970s. His manic periods were easily identified by long and stimulating phone calls in his characteristically deep-throated Italian accent, which would come at any time of the day or night. Indeed, the last communication I had from him, 10 days before he died, was an offer of his lung ascites to culture his tumor cells and a request for references on the genetics of lung cancer.

Ceppellini was undoubtedly one of the most charismatic and original thinkers in the fields of human genetics and immunology in the second half of the 20th century. He is, however, in my view, not sufficiently appreciated for his many scientific contributions. It is gratifying, therefore, to see his memory sustained by the European Federation of Immunogenetics (EFI) annual Ceppellini award lecture, first given by Jon van Rood very shortly after his death, and the stimulating Ceppellini advanced courses in immunology organized by the “Scuola Superiore d' Immunologia Ruggero Ceppellini”.

## Author Contributions

The author confirms being the sole contributor of this work and has approved it for publication.

### Conflict of Interest Statement

The author declares that the research was conducted in the absence of any commercial or financial relationships that could be construed as a potential conflict of interest.

## References

[1] Bodmer In memoriam: Ruggero Ceppellini 1917–1988. Immunogenetics. (1989) 29:145–7. 2647630 10.1007/BF00373638

[2] Ceppellini R Dunn LC Turri M. An interaction between alleles at the Rh locus in man which weakens the reactivity of the Rh(0) factor (D). Proc Natl Acad Sci USA. (1955) 41:283–8. 16589665 10.1073/pnas.41.5.283 PMC528079

[3] Ceppellini R Siniscalco M Una nuovaipotesi genetica per il sistema lewis-secretore esuoi riflessi nei riguardi di alcune evidenze di linkage con altri loci. Rivista dell'Istituto Sieroterapico Italiano. (1955) 30:431–45. 13298490

[4] Ceppellini R Dunn LC Innella F Immunogenetica II: analisi genetica formale dei caratteri Lewis con particolare riguardo alla natura epistatica della specificità serologica Leb. Folia Heredit Pathol. (1959) VIII:261–96.

[5] Ceppellini R Negative correlation between altitude above sea level and incidence of thalassemia in four Rardinian villages. Cold Spring Harb Symp Quant Biol. (1955) 2:252.

[6] Grubb R. Agglutination of erythrocytes coated with incomplete anti-Rh by certain rheumatoid arthritic sera and some other sera; the existence of human serum groups. Acta Pathol Microbiol Scand. (1956) 39:195–7. 13372249

[7] Ceppellini R Genetica delle Immunoglobuline. Atti Associazione Genetica Italiana. (1967) 12:3–131.

[8] Dausset J Brecy H. Identical nature of the leucocyte antigens detectable in monozygotic twins by means of Iso-Leuco agglutinins. Nature. (1957) 180:1430. 13493557 10.1038/1801430a0

[9] Dausset J Iso-leuco-anticorps. Acta Haematol. (1958) 20:156–66. 13582558 10.1159/000205478

[10] Terasaki P History of HLA: Ten Recollections. Los Angeles, CA: UCLA Tissue (1990).

[11] Cavalli-Sforza LL Bodmer WF (1971). The Genetics of Human Populations. San Francisco, CA: W.H. Freeman & Co, Dover Publications.

[12] Ceppellini R Curtoni ES Mattiuz PL Leigheb G Visetti M Colombi A Survival of test skin grafts in man: effect of genetic relationship and of blood groups incompatibility. Ann. N.Y. Acad. Sci. (1966) 129:421–45.

[13] van Rood JJ van Leeuwen A Schippers R Ceppellini PL Mattiuz S Curtoni S. Leukocyte groups and their relationship to homo transplantation. Ann NY Acad Sci. (1966) 129:467–72.

[14] Ceppellini R Bigliani S Curtoni ES Leigheb G Experimental allotransplantation in man. II. The role of A1, A2, and B antigens. 3. Enhancement by circulating antibody. Transplant. Proc. (1969) 1:390. 4944246

[15] Ceppellini R Bonnard GD Coppo F Miggiano VC Pospisil M Curtoni ES. Transplantation antigens: introductory symposium. Mixed leukocyte cultures and HL-A antigens. I. Reactivity of young fetuses, newborns and mothers at delivery. Transplant.Proc. (1971) 3:63. 4106331

[16] Bach FH Amos DB Hu-l: Major histocompatibility locus in man. Science. (1967) 156:1506. 4887739 10.1126/science.156.3781.1506

[17] Contu L Carcassi C Orrù S Mulargia M Arras M Boero R. HLA-B35 frequency variations correlate with malaria infection in Sardinia. Tissue Anti. (1998) 52:452–61. 9864035 10.1111/j.1399-0039.1998.tb03072.x

[18] Mattiuz PL Ihde D Piazza A Ceppellini R Bodmer WF In: Terasaki PI, editor. Histocompatibility Testing. Copenhagen: Munksgaard (1970). p. 193–205.

[19] Ceppellini R Siniscalco M Smith CA. The estimation of gene frequencies in a random-mating mating population. Ann Hum Genet. (1955) 20:97–115. 13268982 10.1111/j.1469-1809.1955.tb01360.x

